# Ultrafast Jahn‐Teller Photoswitching in Cobalt Single‐Ion Magnets

**DOI:** 10.1002/advs.202206880

**Published:** 2023-05-17

**Authors:** Sophie E. Canton, Mykola Biednov, Mátyás Pápai, Frederico A. Lima, Tae‐Kyu Choi, Florian Otte, Yifeng Jiang, Paul Frankenberger, Martin Knoll, Peter Zalden, Wojciech Gawelda, Ahibur Rahaman, Klaus B. Møller, Christopher Milne, David J. Gosztola, Kaibo Zheng, Marius Retegan, Dmitry Khakhulin

**Affiliations:** ^1^ European XFEL Holzkoppel 4 22869 Schenefeld Germany; ^2^ Department of Chemistry Technical University of Denmark Kongens Lyngby DK‐2800 Denmark; ^3^ Wigner Research Centre for Physics P.O. Box 49 Budapest H‐1525 Hungary; ^4^ XFEL Division Pohang Accelerator Laboratory Jigok‐ro 127‐80 Pohang 37673 Republic of Korea; ^5^ Departamento de Química Universidad Autónoma de Madrid Madrid 28049 Spain; ^6^ IMDEA‐Nanociencia Calle Faraday 9 Madrid 28049 Spain; ^7^ Faculty of Physics Adam Mickiewicz University Poznan 61‐614 Poland; ^8^ Chemical Physics and NanoLund Lund University Box 124 Lund 22100 Sweden; ^9^ Center for Nanoscale Materials Argonne National Laboratory 9700 South Cass Avenue Lemont IL 60439 USA; ^10^ European Synchrotron Radiation Facility 71 Avenue des Martyrs Grenoble 38000 France

**Keywords:** Jahn‐Teller effect, photoswitching, single‐ion‐magnets, XFEL science

## Abstract

Single‐ion magnets (SIMs) constitute the ultimate size limit in the quest for miniaturizing magnetic materials. Several bottlenecks currently hindering breakthroughs in quantum information and communication technologies could be alleviated by new generations of SIMs displaying multifunctionality. Here, ultrafast optical absorption spectroscopy and X‐ray emission spectroscopy are employed to track the photoinduced spin‐state switching of the prototypical complex [Co(terpy)_2_]^2+^ (terpy = 2,2′:6′,2″‐terpyridine) in solution phase. The combined measurements and their analysis supported by density functional theory (DFT), time‐dependent‐DFT (TD‐DFT) and multireference quantum chemistry calculations reveal that the complex undergoes a spin‐state transition from a tetragonally elongated doublet state to a tetragonally compressed quartet state on the femtosecond timescale, i.e., it sustains ultrafast Jahn‐Teller (JT) photoswitching between two different spin multiplicities. Adding new Co‐based complexes as possible contenders in the search for JT photoswitching SIMs will greatly widen the possibilities for implementing magnetic multifunctionality and eventually controlling ultrafast magnetization with optical photons.

## Graphical abstract

Combined optical and X‐ray spectroscopy experiments complemented by theoretical calculations reveal the photoinduced Jahn‐Teller photoswitching between two spin multiplicities in a Co(II)‐based molecular complex, which occurs on the femtosecond timescale. Unveiling the associated transient magnetic anisotropy will assist the development of such systems into light‐driven multifunctional single‐ion magnets and qubits.

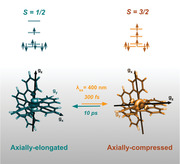

## 1 Introduction

The striking discovery in the early 1990s that anisotropic molecules carrying individual spins **S** can orient preferentially in an external magnetic field **B** and retain their magnetization at low temperature has marked a new dawn in molecular magnetism.^[^ ^1^, ^2^, ^3^, ^4^ ^]^ The subsequent development of single‐molecule magnets (SMMs) and their systematic exploitation in bottom‐up designs has matured into an active multidisciplinary field, which is bolstering rapid progress in switching and sensing nanotechnologies.^[^ ^5^, ^6^, ^7^, ^8^, ^9^ ^]^ Within a quantum‐mechanical framework, the magnetic properties of SMMs are described based on the zero‐field (Z‐F) hamiltonian H

(1) $$\;H=DS_{z}^{2}+E\left(S_{x}^{2}+S_{y}^{2}\right)$$

where *D* and *E* are the axial and the rhombic anisotropy, S_i_ (*i* = *x,y,z*) are the projections of the spin vector on the respective axis *i* with S being the value of the total spin. In general, SMMs with *S* > 1 present a barrier to magnetization reversal,^[^ ^10^, ^11^, ^12^ ^]^ while SMMs with *S* < 1 undergo light‐induced magnetization.^[^ ^13^, ^14^, ^15^ ^]^ For practical applications, the amplitude of the magnetization, its relaxation time and its blocking temperature should be maximized.^[^ ^16^, ^17^, ^18^ ^]^ The possibilities of augmenting the magnetization by simply increasing the number of spins N are intrinsically limited, due to the fact that the spins cannot be easily controlled in SMMs of high nuclearity^[^ ^19^ ^]^ and that *D* only scales as *S* (rather than *S^2^*).^[^ ^20^, ^21^ ^]^ In contrast, mononuclear SMMs first reported in 2010, also known as single‐ion magnets (SIMs), display large *D* values with the added features of steric control and minimal size.^[^ ^22^, ^23^, ^24^, ^25^, ^26^ ^]^

In order to meet the growing demands of nanoscience and quantum information technology, research efforts are now targeting new generations of SIMs possessing adaptive opto‐electromagnetic properties.^[^ ^27^, ^28^, ^29^, ^30^, ^31^, ^32^, ^33^ ^]^ A promising line of research relies on integrating the bistability afforded by the low‐spin (LS) and high‐spin (HS) states of mononuclear transition metal complexes with d^4^–d^7^ electronic configurations, a phenomenon known as spin crossover (SCO) when the spin‐state transition is e.g., thermally induced. Proof‐of‐principle applications utilizing concurrently the SIM and SCO magnetic bistabilities have started to emerge.^[^ ^34^, ^35^, ^36^, ^37^, ^38^ ^]^ However, achieving similar synergy for fast switching is still conditional to extending the control of the photoinduced spin‐state transition down to the femtosecond timescale.^[^ ^39^, ^40^, ^41^ ^]^ A fundamental step toward this goal requires identifying the factors that determine the characteristics of the ultrafast switching dynamics at the atomic level.

In this work, femtosecond transient optical absorption spectroscopy (OAS) and X‐ray emission spectroscopy (XES) are combined to track the photoinduced spin‐state switching of [Co(terpy)_2_]^2+^ (terpy = 2,2′:6′,2″‐terpyridine)^[^ ^42^, ^43^, ^44^ ^]^ in solution, which is a prototypical benchmarking complex in SCO and SIM research. With the support from density functional theory (DFT), time‐dependent DFT (TD‐DFT) and multireference quantum chemical calculations, the analysis of the measurements delivers insights into the coupled electronic and geometric changes that drive the photoswitching process.

## 2 Results

### 2.1 Probing the Photoinduced Spin‐Switching Dynamics

**Figure 1:**
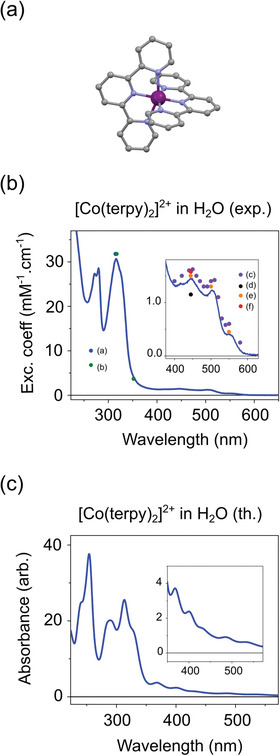
a) COSMO‐DFT structure for the ground‐state of [Co(terpy)_2_]^2+^ in H_2_O. The Co, N, and C atoms are displayed respectively in purple, blue, and gray. The hydrogen atoms are omitted for clarity. b) Experimental extinction coefficient of [Co(terpy)_2_]^2+^ in H_2_O, along with the previously published values (The references (a) to (f) are given in Section S.I.1, Supporting Information). c) Theoretical UV–vis spectrum of [Co(terpy)_2_]^2+^ in H_2_O.

The mononuclear complex [Co(terpy)_2_]^2+^ is built out of two rigid tridentate ligands that coordinate a d^7^ Co(II) center (Section S.I.1, Supporting Information). The crystallographic structure reveals a molecular geometry of distorted D_2d_ symmetry with two inequivalent metal‐ligand bond lengths Co—N_axial_ and Co—N_equatorial_.^[^ ^45^, ^46^, ^47^ ^]^ The structure produced by DFT optimization using a conductor‐like model (COSMO) for the solvent exhibits the same characteristics (**Figure** **1**a) (see Section S.I.2, Supporting Information). The electronic ground state is a LS doublet, where Co(II) has a spin *S* = 1/2.

Figure 1b displays the extinction coefficient of [Co(terpy)_2_]^2+^ in water (H_2_O) over the UV–vis range, along with the previously published values. The corresponding references (a) to (f) are given in Section S.I.1 in the Supporting Information.

The spectrum is dominated by a large band centered at 315 nm, while three weak peaks are observed on the flank at 447, 505, and 555 nm, in agreement with previous reports (Section S.I.1, Supporting Information). Figure 1c shows the theoretical UV–vis spectrum of [Co(terpy)_2_]^2+^ in H_2_O calculated with TD‐DFT (see Section S.I.2, Supporting Information), where the absorbance in the 400–600 nm wavelength range is dominated by metal‐to‐ligand charge transfer (MLCT) transitions.

**Figure 2:**
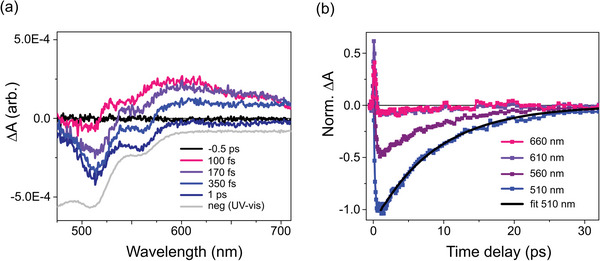
a) Transient optical absorption spectra for [Co(terpy)_2_]^2+^ in H_2_O excited at 400 nm, for selected time delays Δ*t*. b) Normalized kinetics at 660 nm (magenta), 610 nm (violet), 560 nm (purple), and 510 nm (blue) along with its single‐exponential fit (black).

The photoinduced dynamics are first probed with femtosecond transient OAS in the visible. **Figure** **2**a displays the spectral evolution on the sub‐picosecond timescale at selected time delays, following excitation at 400 nm (see Section S.I.3, Supporting Information). A broad positive transient signal appears within the instrument response function (IRF) of 165 fs full width half‐maximum (fwhm). It decays over a few hundreds of femtoseconds to form a bleach signal that resembles closely the negative of the steady‐state UV–vis spectrum (gray line). Figure 2b shows the kinetics of the bleach signal at 510 nm after normalization of its minimum to −1. A lifetime of ≈6.4 ± 0.4 ps is extracted for the lowest‐excited metastable state by fitting this kinetics with a single exponential decay (see Section S.I.3, Supporting Information).

**Figure 3:**
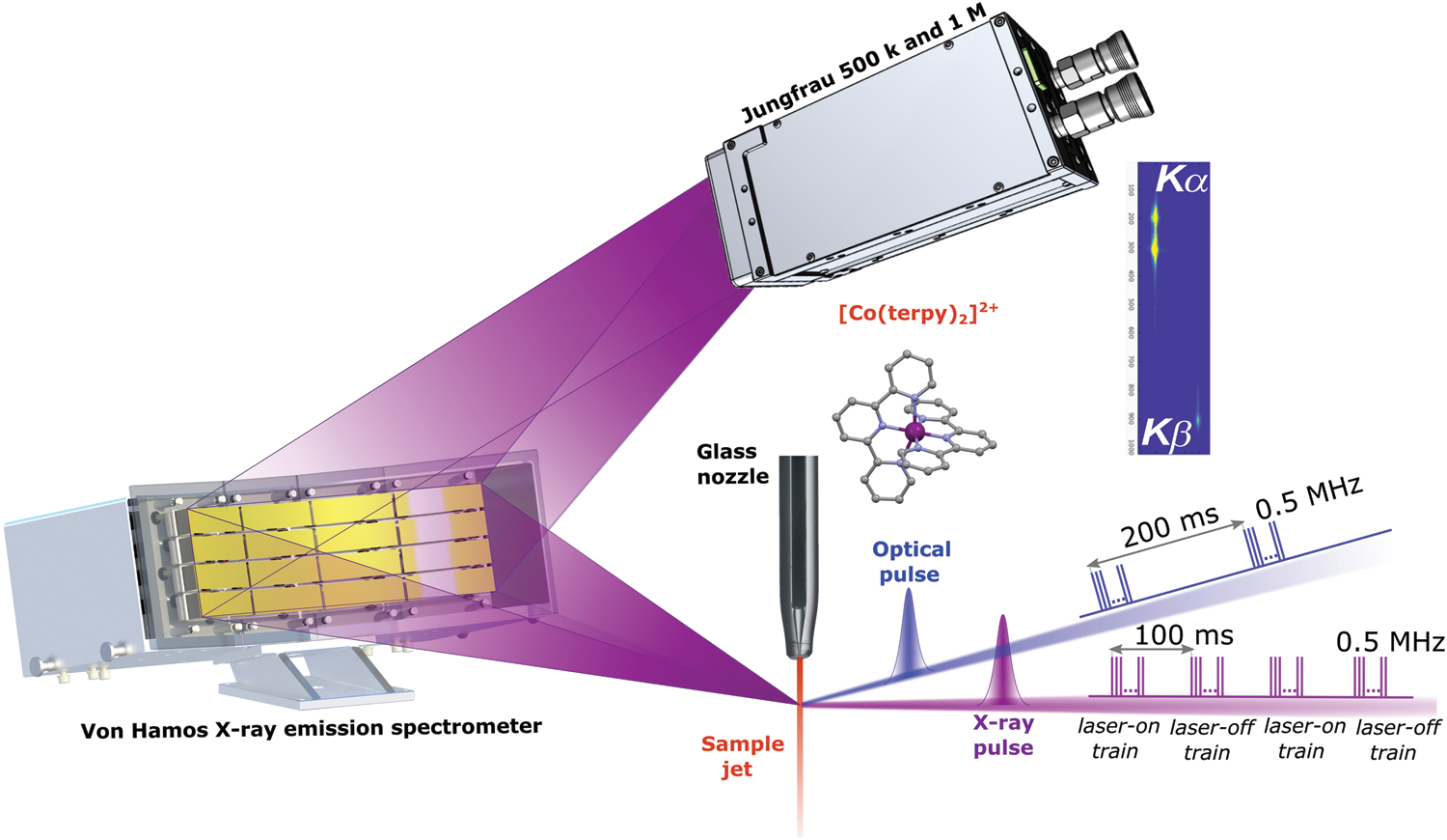
Experimental setup for femtosecond X‐ray emission spectroscopy at the FXE Instrument of the European XFEL.

The photoinduced dynamics are then monitored with femtosecond transient XES at the femtosecond X‐ray experiments (FXE) instrument of the European XFEL facility^[^ ^48^, ^49^ ^]^ in order to track the dynamics with spin sensitivity and to assign the spin multiplicity of the excited‐state.^[^ ^50^, ^51^, ^52^ ^]^ The experimental setup is shown in **Figure** **3**. The data acquisition and the data analysis procedures are described in Section S.I.4 in the Supporting Information. The K*α*_1,2_ and K*β* X‐ray emission lines originate respectively from the radiative decay 2p_1/2,3/2_ → 1s and 3p_1/2,3/2_ → 1s, subsequent to core ionization from the 1s level. The lineshapes depend primarily upon the spin multiplicity of the Co center.^[^ ^52^, ^53^ ^]^

**Figure 4:**
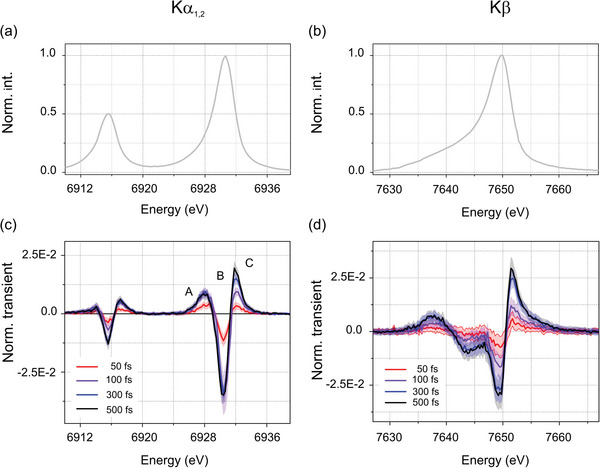
a) Static K*α*_1,2_ (b) and K*β* spectra for [Co(terpy)_2_]^2+^ in H_2_O, normalized to their intensity maxima. c) Normalized K*α*_1,2_ and d) K*β* transient difference profiles observed for pump–probe delays Δ*t* of 50, 100, 300, and 500 fs after optical excitation at 400 nm. The shaded areas around the thin lines indicate the experimental error bars.

**Figure** **4**a,b show the steady‐state K*α*_1,2_ and K*β* lines of [Co(terpy)_2_]^2+^ in H_2_O. The profiles are characteristic of Co(II) doublets. Figure 4c,d displays the K*α*_1,2_ and K*β* transient difference signals observed at fixed pump–probe delays Δ*t* of 50, 100, 300, and 500 fs after optical excitation at 400 nm. The K*α*_1_ transient difference lineshape of [Co(terpy)_2_]^2+^ can be decomposed into two positive bands (noted A and C) and a negative band B. The K*β* transient difference lineshape of [Co(terpy)_2_]^2+^ presents a complex profile with a K*β*’ feature around 7637 eV, which is a known fingerprint of the quartet state.^[^ ^53^ ^]^

The K*α*_1_ reference traces obtained by taking the difference between the static spectra of [Co(bpy)_3_]^2+^ (quartet, *S* = 3/2) and [Co(terpy)_2_]^2+^ (doublet, *S* = 1/2) collected at FXE are shown in Section S.I.4 in the Supporting Information. The agreement between the reference traces and the transient difference spectra at 500 fs demonstrates that photoexcitation triggers a spin‐state transition from the LS doublet (*S* = 1/2) ground state to a metastable HS quartet (*S* = 3/2) state. The relative scaling factor yields an excited state fraction of ≈25 ± 5% at 500 fs. The slight mismatch that can be noted between the transient and the reference difference spectra could be ascribed to the differences in solvents and in coordination (bidentate versus tridentate).

**Figure 5:**
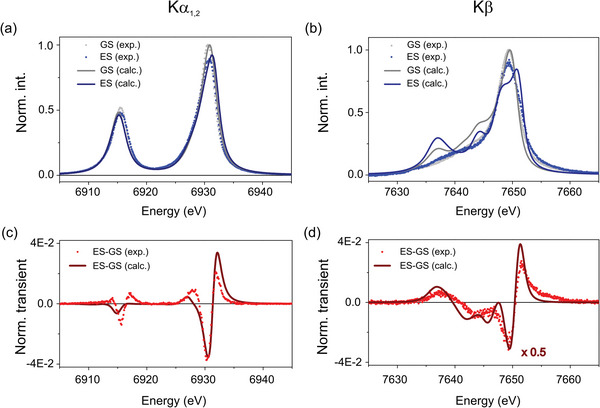
a) Experimental and calculated K*α*_1,2_ spectral lines of the quartet excited‐state (blue dots and blue line respectively) and of the doublet ground‐state (gray dots and gray line respectively). b) Experimental and calculated K*β* lines of the quartet excited‐state with laser (blue dots and blue line respectively) and of the doublet ground‐state (gray dots and gray line respectively). c) Experimental and calculated K*α*_1,2_ difference spectra. d) Experimental and calculated K*β* difference spectra. The amplitude of the calculated spectrum is scaled by 0.5 to facilitate the visual comparison. The pump–probe delay associated with all experimental traces is Δ*t* = 500 fs.

**Figure** **5**a,b shows the experimental and the modeled K*α*_1,2_ and K*β* profiles of the doublet and quartet species for [Co(terpy)_2_]^2+^. The spectra are calculated with the Crispy graphical user‐interface^[^ ^54^ ^]^ for the Quanty library.^[^ ^55^ ^]^ The systems are described by a semi‐empirical Hamiltonian that includes atomic and crystal‐field interactions, similar to the one employed to model the XES spectra of iron compounds.^[^ ^56^ ^]^ The values of the parameters used in the calculations are presented in Section S.I.5 in the Supporting Information. Figure 5c,d displays the calculated K*α*_1,2_ and K*β* transient difference profiles, which are in good agreement with the experimental measurements. The mismatch can be partly associated with the neglect of covalency, which is revealed by the delocalization of the spin density observed in the spin‐up and spin‐down unrestricted Kohn‐Sham orbitals of the doublet ground state and the lowest quartet excited state (figure in Section S.I.2, Supporting Information).

The transient K*α*_1_ difference profile acquired at a nominal pump–probe delay Δ*t* of 50 fs presents A and C bands of comparable intensities (Figure 4c). This lineshape is distinct from the profile ascribed to the doublet‐quartet transition observed for a pump–probe delay Δ*t* of 500 fs, where the amplitude of C is larger than the one of A. Therefore, the photoinduced spin‐state switching between the doublet and the quartet state proceeds through an intermediate step. However, the lineshape at Δ*t* of 50 fs cannot be reproduced by any admixture of the doublet‐MLCT profile (see Section S.I.6, Supporting Information), so that the initially populated MLCT is not observed in the present XES measurements with a ≈115 fs fwhm IRF.

**Figure 6:**
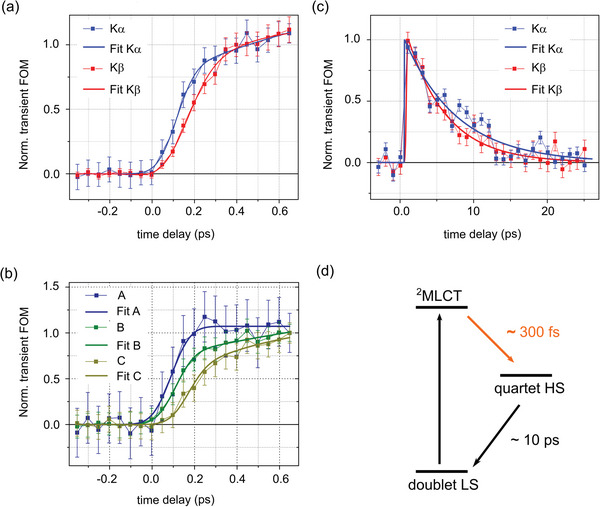
a) K*α*_1_ and K*β* kinetics on the sub‐ps timescale, monitoring the quartet HS formation. b) Emission‐energy‐resolved kinetics across the K*α*_1_ line on the sub‐ps timescale and the best fits resulting from Model III (see main text). The areas A (blue), B (green), and C (light green) are indicated in Figure 4c. c) K*α*_1_ (blue) and K*β* (red) kinetics tracking the lifetime of the quartet HS state. For panels (a) to (c), the data points at each pump–probe delay are given by the sum of the absolute values of the transient difference signal over the emission energy ranges specified in the main text. d) Schematics of the photocycle for [Co(terpy)_2_]^2+^ in H_2_O, with a non‐adiabatic formation on the 300 fs timescale (orange arrow) and a decay on the sub 10 ps timescale (black arrow).

Previous studies on six‐coordinated Co(II) complexes have established that the correlated changes in the width and maximum intensity of the K*α*_1_ line reflect the evolution of the spin‐state at the Co center.^[^ ^52^, ^53^ ^]^ Here, the summed absolute values of the integrated areas under the transient difference signals are taken as figure of merit (FOM) and followed as a function of pump–probe time delay. **Figure** **6**a focuses on the K*α*_1_ and K*β* XES kinetics acquired simultaneously over the first picosecond following photoexcitation. These traces capture intrinsic timescales in the dynamic formation of the quartet HS state. Fixing the fwhm IRF to the standard ≈115 fs of liquid‐phase chemistry measurements at the FXE instrument,^[^ ^49^ ^]^ the rise times are 270 ± 80 fs for K*α*_1_ and 320 ± 400 fs for K*β* (Section S.I.7, Supporting Information). Considering that the K*α*_1_ and K*β* traces are acquired simultaneously, the offset in *t*_0_ of ≈50  fs is inherent to the intermediate stage in the early dynamics. This point is further confirmed by Figure 6b, which displays the three emission‐energy‐resolved kinetics across the K*α*_1_ line, after normalization to the last data point. These traces correspond to the temporal evolution of the FOM for bands A, B, and C. The positive transient A clearly rises faster than the negative B and the positive C. These three kinetics can be fitted with three different models (see details in Section S.I.7, Supporting Information). In Model I, the three bands rise with the same time‐constant *τ*, but start from different initial times *t_0,i_*, *i* = 1,2,3. In Model II, the three bands start from the same initial time *t*_0_, but rise with three different time‐constants *τ_i_, i* = 1,2,3. In Model III, the three *t_0,i_* and the three *τ_i_* are free to vary. The numerical details, the best‐fit parameters and the goodness of fit are summarized in Section S.I.7 in the Supporting Information. With six parameters, Model III appears better suited for describing the early non‐adiabatic dynamics as expected (Section S.I.7, Supporting Information). The delay in *t_0,i_* might be associated with the early charge‐transfer (CT) character of the Franck‐Condon state, while differences in *τ_i_* may be due to competing intersystem crossing (ISC) and structural dynamics. The elaboration of a complete model for the non‐adiabatic formation of the quartet state requires the acquisition of extended datasets in several solvents of different properties (e.g., polarity). On a longer timescale, the K*α*_1_ and K*β* kinetics shown in Figure 6c decay with a time‐constant of 7.5 ± 1.3 and 5.4 ± 0.6 ps, which are close to the lifetime obtained from the transient OAS measurements (Figure 2b). The time constants of the non‐adiabatic formation of the quartet HS and its decay are both indicated in the schematic photocycle shown in Figure 6d.

### 2.2 Characterizing the Photoinduced Spin‐Switching Mechanism

**Figure 7:**
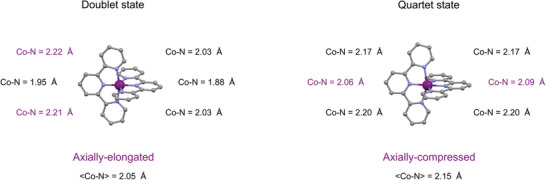
DFT optimized structures for the axially elongated doublet and the axially compressed quartet species. The individual Co—N bond lengths and the average <Co—N> bond lengths are indicated.

The ultrafast OAS and XES measurements first demonstrate that efficient doublet‐quartet spin‐state switching takes place on the sub‐picosecond timescale (Figure 2 and Figure 6). Close inspection of the geometric parameters presented in **Figure** **7** shows that the DFT structure of the lowest doublet state D_0_ is axially elongated, since two Co—N_equatorial_ (2.22 and 2.21 Å) are longer than the four other Co—N bonds, namely the two Co—N_equatorial_ (2×2.03 Å) and two Co—N_axial_ bonds (1.95 and 1.88 Å). In contrast, the DFT structure of the lowest quartet state Q_1_ is axially compressed, since the two Co—N_axial_ (2.06 and 2.09 Å) are shorter than the four other Co—N bonds, namely the four Co—N_equatorial_ bonds (2×2.17 and 2×2.20 Å).

**Figure 8:**
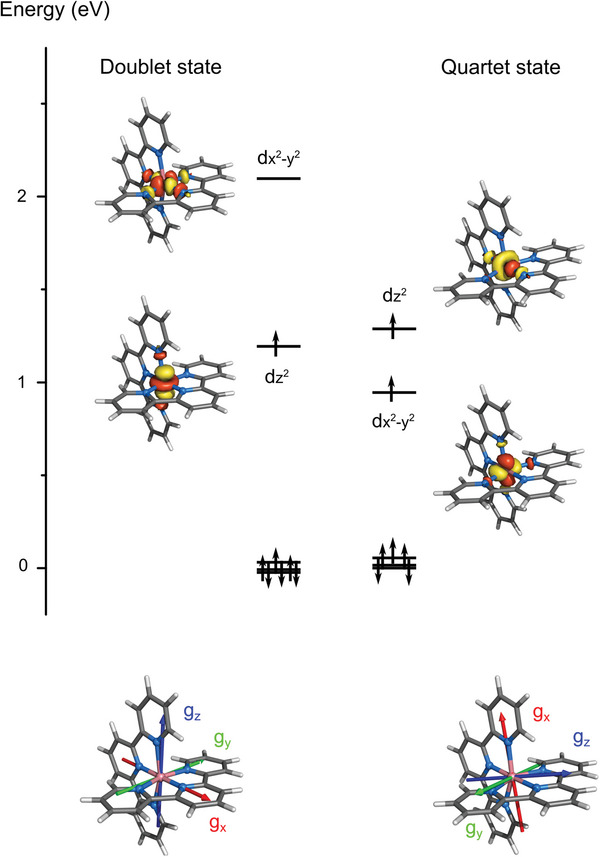
SA‐CASSCF orbitals from AILFT calculations for the doublet and quartet structures obtained from the DFT optimizations. The *g_x_, g_y_*, and *g_z_* components of the g‐tensor are shown in red, green, and blue.

Orca ab initio ligand field theory (AILFT) calculations based on complete active space self‐consistent field and N‐electron valence state second‐order perturbation theory (CASSCF/NEVPT2) wave‐function methods^[^ ^57^ ^]^ were used to obtain the ligand‐field parameters for the structures of the doublet and quartet species optimized with DFT (Section S.I.2, Supporting Information). The state‐average CASSCF (SA‐CASSCF) d‐orbitals are shown in **Figure** **8**. The relative energetic ordering of $d_{z^{2}}$ and $d_{x^{2}-y^{2}}$ goes from $d_{z^{2}}<d_{x^{2}-y^{2}}$ in the doublet state to $d_{x^{2}-y^{2}}<d_{z^{2}}$ in the quartet state. For the doublet state, the $d_{z^{2}}$ orbital aligns along the two Co—N_equatorial_ bonds of one terpy ligand, while for the quartet state, the $d_{z^{2}}$ orbital aligns along the two Co—N_axial_ bonds involving the two terpy ligands. In other words, the *z*‐axis, which is imposed by the local ligand‐field and lies along the largest component of the g‐tensor (blue arrow in Figure 8) flips from the direction determined by the two longest bonds in the axially elongated doublet species to the two shortest bonds in the axially compressed quartet species.

Therefore, the first key result of this study is that photoexciting [Co(terpy)_2_]^2+^ in H_2_O induces an ultrafast spin‐state transition from a tetragonally elongated doublet state to a tetragonally compressed quartet state, which is accompanied by a reorientation of the principal axes of the g‐tensor. The geometric and magnetic anisotropy axes of the molecular complex are both efficiently switched with light.

In addition, the ultrafast OAS and XES measurements demonstrate that the photoinduced spin‐state transition proceeds through an intermediate step. With a d^7^ electronic configuration in a quasi‐octahedral geometry, the LS ground state of [Co(terpy)_2_]^2+^ is subject to the Jahn‐Teller (JT) effect that imposes a net distortion approximated by a double axial bending.^[^ ^58^, ^59^ ^]^ Impulsive promotion of a d electron to the ligand via MLCT creates a transient effective d^6^ Co(III) center and excites synchronously the coupled modes involved in this distortion, namely the radial breathing mode and the angular pincer mode.^[^ ^58^, ^59^, ^60^, ^61^ ^]^ Therefore, the early non‐adiabatic dynamics are governed by a complex interplay between JT dynamics and ISC. Nevertheless, under the simplifying assumption of a sequential decay, the experimental observation of an optically bright stage in the transient OAS measurements with delayed onsets in the energy‐dependent XES kinetics highlights the prolonged participation of the doublet manifold in the formation of the quartet state.

**Figure 9:**
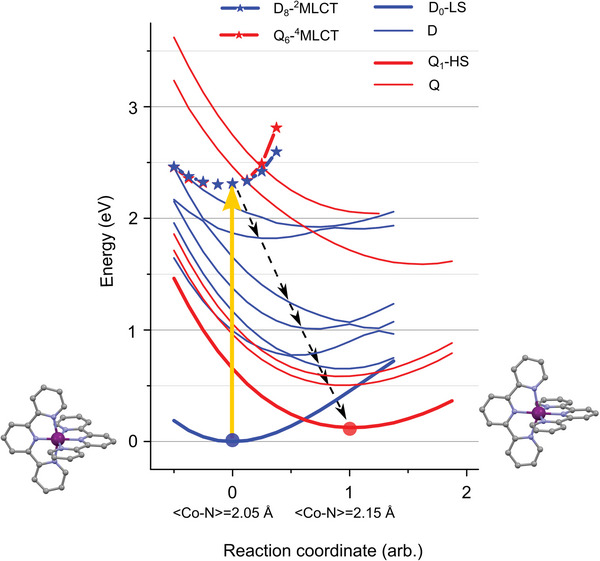
TD‐DFT potential energy curves (PECs) along the 1D reaction coordinate that corresponds to the linear interpolation from the lowest doublet structure to the lowest quartet DFT structure shown in Figure 7. Their LS/HS PECs are indicated by the thick blue/red lines. The excited doublet/quartet MC states are indicated with thin blue/red lines. The states with MLCT character are displayed with starred lines. The yellow arrow represents the optically allowed Franck‐Condon transition. The black dashed arrows illustrate a possible sequential pathway (see main text).

**Figure** **9** displays the potential energy curves (PECs) constructed from TD‐DFT calculations using the linear geometric interpolation from the D_0_ to the Q_1_ structures (Figure 7) as multimodal 1D reaction coordinate (RC). The numerical parameters used for the calculations are given in Section S.I.2 in the Supporting Information. For the lowest doublet state with an average Co—N bond <Co—N> of 2.05 Å, RC = 0. For the quartet state with a <Co—N> of 2.15 Å, RC = 1. As a first step, the vibronic coupling (VC) and the spin–orbit coupling (SOC) are discarded in order to keep the calculations tractable. The excited states of doublet (D) and quartet (Q) multiplicities are represented by thin blue and red lines respectively. The structure corresponding to the lowest doublet‐quartet intersection is displayed in Section S.I.8 in the Supporting Information.

Upon photoexcitation, the molecular complex is promoted from its doublet ground state to the doublet MLCT state through an optically allowed transition (yellow arrow). Both states present similar shapes of PECs in the Franck‐Condon region. The conversion from the doublet to the quartet multiplicity is induced by ISC. Although the inclusion of VC and SOC is expected to modulate the transitions across the excited‐state landscape, the PECs in Figure 9 suggest a sequential pathway, where the non‐adiabatic JT dynamics take place in the doublet metal‐centered (MC) manifold and could mediate the ISC. An approximate path is indicated by the black dashed arrows.

Therefore, the second key result of this study is that the photoinduced spin‐state transition in [Co(terpy)_2_]^2+^ in H_2_O is driven by ultrafast JT switching.

Unraveling experimentally the non‐adiabatic dynamics driven by the interplay between JT effect, VC and SOC will require more detailed systematic investigations of the optically bright and optically dark ultrafast dynamics with spin‐sensitivity in rationally designed families of Co complexes, some of which will be presented elsewhere. Modeling theoretically the photoinduced spin‐state switching will rely on introducing VC and SOC in the Franck‐Condon MLCT transition and throughout the MC relaxation.^[^ ^62^ ^]^

## 3 Discussion

SIMs that sustain JT photoswitching (JT‐SIMs) are of practical interest for optimizing the optical manipulation of qubits and the control schemes of magnetic anisotropy in quantum information technologies.^[^ ^27^, ^63^, ^64^, ^65^, ^66^ ^]^ The JT effect is also recognized as a mechanism that efficiently couples the molecular electronic degrees of freedom to the nuclei vibrations in the surrounding lattice.^[^ ^67^, ^68^, ^69^, ^70^ ^]^ While the JT‐SIMs reported so far have been mostly built around Mn and Cu,^[^ ^71^, ^72^, ^73^, ^74^ ^]^ the present work demonstrates that Co complexes can also be considered for developing innovative multifunctional JT‐SIMs based on their specific properties. Having a d^7^ electronic configuration requiring the populations of orbitals with e_g_‐like character, Co(II) complexes are invariably subject to the JT distortion, so that the process reported here is expected to be generally at play upon photoexcitation.

**Figure 10:**
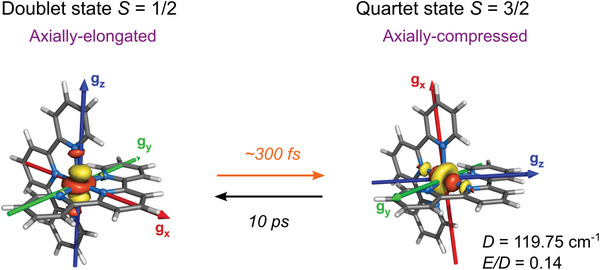
Summary of the JT‐photoswitching characteristics for [Co(terpy)_2_]^2+^ in H_2_O. Geometric structures (with distortion), $d_{z^{2}}$ orbitals, and g_*x*_, g_*y*_, g_*z*_ components of the g‐tensors for the doublet and quartet species. The values of the axial and transverse anisotropies *D* and *E* are indicated for the HS species. The time scales for the formation of the quartet state upon photoexcitation of the doublet state (orange arrow) and for the decay from the quartet state back to the doublet state (black arrow) are also displayed.

Summarizing the experimental and theoretical findings, **Figure** **10** displays the geometric structures (with distortion) and the orientations of the $d_{z^{2}}$ orbital for the doublet and quartet species, along with their magnetic attributes, including the g*_x_*, g*_y_*, g*_z_* components of the g‐tensors, and the values of the theoretical axial and transverse magnetic anisotropy *D* (119.75 cm^−1^) and *E* (0.14 × 119.75 = 16.76 cm^−1^) for the quartet state. It should be recalled that the Z‐F Hamiltonian cannot describe species with *S* < 1, such as here [Co(terpy)_2_]^2+^ in its doublet ground state. The timescales (formation and decay) characterizing the photoswitching process between the doublet and quartet states for [Co(terpy)_2_]^2+^ in H_2_O are also indicated in Figure 10. They can be compared to the ones reported for related spin crossover molecular complexes in Section S.I.9 in the Supporting Information. Although the photoswitching parameters are not optimal in the simple [Co(terpy)_2_]^2+^ complex, the present study nevertheless highlights a path for developing multifunctional JT‐SIMs. Since Co(II) complexes in the LS state can display field‐induced magnetization and qubit behavior, while Co(II) complexes in the HS state can act as SIMs, future synthesis protocols could produce single Co(II)‐based molecular units that can be toggled with light between two of the most important functionalities required for applications in quantum information technologies, with high responsivity to alterations in the environment.

Moreover, whereas the JT photoswitching observed in Mn‐based JT‐SIMs takes place within the same spin multiplicity (e.g., quintet through ^5^B_1g_ → ^5^A_1g_ transition),^[^ ^71^, ^72^ ^]^ the flipping of the anisotropy axis in Co‐based JT‐SIMs involves two states of different spin multiplicities in addition to the very short‐lived intramolecular charge‐transfer step at the Franck‐Condon point. In contrast to most Fe(II) complexes, the spin‐state transition in Co(II) complexes appears inherently coupled to large tetragonal or trigonal deformations at the molecular level. The early vibrational coherence triggered by the impulsive optical excitation should then be strongly influenced by SOC, VC, and conical intersections.^[^ ^75^ ^]^

Although the understanding of the relationships between the molecular structure and the magnetic anisotropy of Co SIMs has advanced sufficiently to enable rational design,^[^ ^76^, ^77^ ^]^ similar insight has yet to be gained for photoexcited Co SIMs. It should be noted that a general model able to treat the SCO phenomenon and the JT effect in Co(II) complexes is not yet available, a fact which renders the description of the photoinduced spin‐state transition all the more challenging. Building up this knowledge can now be achieved by identifying robust spin‐resolved correlations between the photoinduced changes of the electronic and geometric structures. A particular focus should be directed at elucidating the connection between the distortions of the molecular framework,^[^ ^58^, ^59^, ^60^ ^]^ the resulting changes in magnetic anisotropy and the prolongation of the lifetime for the metastable spin‐state, eventually as a function of temperature and applied magnetic field strength and frequency. Toward this goal, exploring the photoinduced dynamics of Co(II) complexes in the solution phase across the vast parameter space afforded by their versatile coordination chemistry and their sensitivity to the surroundings stands as a clear starting point.

## 4 Conclusion

In conclusion, ultrafast measurements using optical absorption spectroscopy and X‐ray emission spectroscopy establish that photoexcitation of [Co(terpy)_2_]^2+^ in the solution phase induces ultrafast Jahn‐Teller photoswitching between two different spin multiplicities. This finding significantly expands the pool of complexes where multifunctionalities based on impulsive spin‐state transition and SIM behavior can be tailored, with great prospects for dual tunability arising from the interplay between the SCO temperature and the magnetization blocking temperature. Systematic investigations on the ultrafast time scale combining optical absorption spectroscopy, X‐ray emission spectroscopy with direct spin‐sensitivity, and X‐ray techniques with direct structural resolving‐power (e.g., X‐ray absorption spectroscopy, diffuse wide‐angle X‐ray scattering or photocrystallography) will uncover how the first coordination sphere (ligands), the second coordination sphere (solvent molecules, counterions), and the long‐range crystal packing forces jointly influence the photoswitching timescale and the lifetime of the metastable spin‐state. With the support from quantum chemistry calculations, these studies will allow bridging the description of the Jahn‐Teller photoswitching across the solution‐solid phase transition, and eventually reveal how the photoinduced changes in spin, electronic and geometric degrees of freedom govern the dynamics of the transient magnetic anisotropy in multifunctional JT‐SIMs.

## Conflict of Interest

The authors declare no conflict of interest.

## Author Contributions

S.E.C. and M.B. contributed equally to this work. The chemical synthesis and characterization were performed by A.R. and K.Z. The femtosecond transient absorption measurements and their analysis were conducted by S.E.C., K.Z., and D.J.G. The femtosecond XES measurements were conducted by F.A.L., T.‐K.C., F.O., Y. J., P.F., M. K., P.Z., S.E.C, M.B., and D.K. The analysis of the transient XES measurements was performed by M.B., S.E.C., and D.K. The DFT and TD‐DFT calculations were performed by M.P. The multireference quantum chemistry calculations and the XES simulations were performed by M.R. S.E.C. wrote the initial paper M.B., M.P., T.‐K.C., K.B.M., D.J.G, K.Z., M.R., and D.K. contributed to the writing, editing, and reviewing. All co‐authors read and approved the final submitted version.

## Supporting information

Supporting Information

## Data Availability

The data that support the findings of this study are available from the corresponding author upon reasonable request.
