# Supplementary material for: Ultrafast Jahn‐Teller Photoswitching in Cobalt Single‐Ion Magnets
Source: Adv Sci (Weinh). 2023 May 17;10(21):2206880. doi: 10.1002/advs.202206880 (PMC10375196; doi:10.1002/advs.202206880)
Supplement: Supplementary file 1 — Supporting Information [file ADVS-10-2206880-s001.pdf]

ADVANCED SCIENCE
Open Access

Supporting Information

for *Adv. Sci.*, DOI 10.1002/advs.202206880

Ultrafast Jahn-Teller Photoswitching in Cobalt Single-Ion Magnets

Sophie E. Canton, Mykola Biednov, Mátyás Pápai, Frederico A. Lima, Tae-Kyu Choi, Florian Otte, Yifeng Jiang, Paul Frankenberger, Martin Knoll, Peter Zalden, Wojciech Gawelda, Ahibur Rahaman, Klaus B. Møller, Christopher Milne, David J. Gosztola, Kaibo Zheng, Marius Retegan and Dmitry Khakhulin**

Table of content

S.I.1 Materials and synthesis

S.I.2 DFT and TD-DFT calculations

S.I.3 Transient optical absorption spectroscopy experiments

S.I.4 Time-resolved X-ray emission spectroscopy experiments

S.I.5 *Ab initio* X-ray emission calculations

S.I.6 Assessing the possible observation of the MLCT in the transient XES measurements

S.I.7 Fitting of the energy-resolved K$\boldsymbol{\alpha}_1$ X-ray kinetics

S.I.8 DFT structure at the crossing between the PECs of the lowest doublet and lowest quartet states

S.I.9 Comparison of the ultrafast photoswitching parameters in spin-crossover molecules

S.I.1 Materials and synthesis

$[Co(terpy)_2](Cl)_2$ and $[Co(terpy)_2](PF_6)_2$ were synthesized following the published procedure (1,2). All reagents were used as received without further purification.

References

(1) Inorg. Chem., 1982, 21, 3013-3022
(2) Dalton Trans., 2005 , 236-237

References for Figure 1b in the main text

(a) blue, Vitello, J. Bio. Inorg. Chem. 6, 578-589 (2001)

(b) green Yandell, Biochimica et Biophysica,4cta. 748 (1983) 263-270 263

(c) purple, Hogg, J. Chem. Soc., 1962, 341-350

(d) black, Judge, Inorganica Chimica Acta1, 68-72, 1967

(e) orange, Kremer, Inorg. Chem. 1982, 21, 3013-3022

(f) red, Beattie, Inorganica Chimica Acta 235 (1995) 245-251

S.I.2 DFT and TD-DFT calculations

All DFT and TD-DFT calculations were carried out using the B3LYP* [reiher2001,reiher2002]/TZVP method as implemented in the ORCA3.0 program package [neese2012]. The B3LYP* exchange-correlation functional was chosen based on its known accuracy for spin-state energetics of transition-metal complexes [papai2013,szemes2020]. Solvation effects were taken into account utilizing the conductor-like screening model (COSMO) [klamt1993] with $\varepsilon = 80.4$, i.e., the dielectric constant of water. Two-electron integrals were approximated by the resolution of identity (RI-J) [neese2003] and chain of spheres (COSX) methods [neese2009].

The Kohn-Sham orbitals for the doublet LS and quartet HS state for $[Co(terpy)_2]^{2+}$ in H_2O are shown in Figure **S1a** and **S1b** below.

The lowest-lying doublet (S = 1/2, LS) and quartet (S = 3/2, HS) states of $[Co(terpy)_2]^{2+}$ were fully optimized by DFT (B3LYP*/TZVP). The plotted unrestricted Kohn-Sham orbitals were extracted from the LS and HS electronic wave functions corresponding to the optimized geometries. Potential energy curves were calculated using TD-DFT based on unrestricted doublet/quartet DFT reference at geometries generated by linear interpolation between the optimized LS and HS structures. In the TD-DFT computations, we used the Tamm-Dancoff approximation (TDA) [hirata1999]. The UV/VIS spectrum was obtained by a 300-state TD-DFT calculation performed at the optimized LS geometry. We broadened the TD-DFT transition intensities using a pseudo-Voigt profile with 1000 cm^{-1} width for both Gaussian and Lorentzian components.

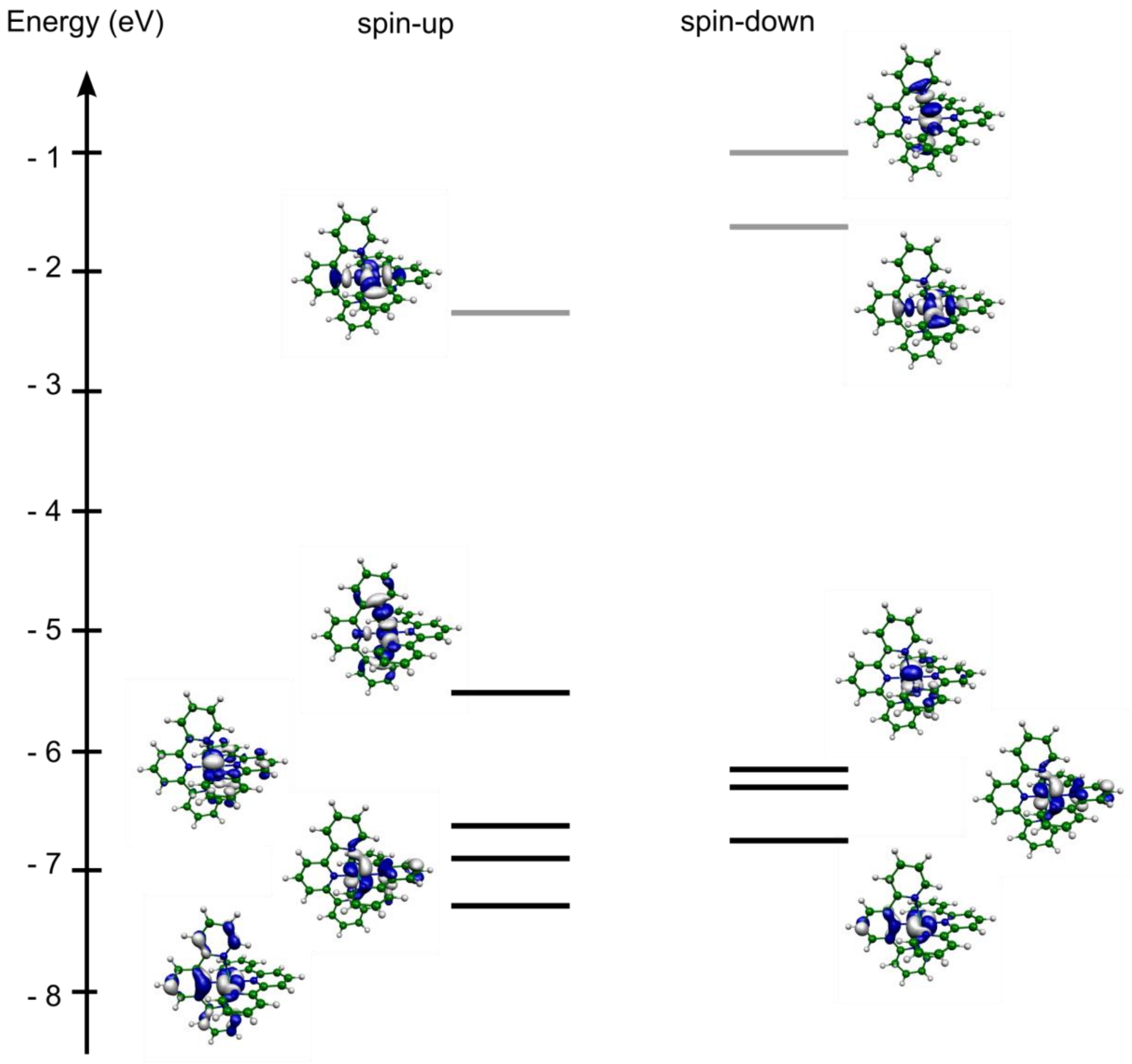

***Figure S1a**. Unrestricted spin-up(alpha)/spin-down(beta) orbitals for the doublet LS state of $[Co(terpy)_2]^{2+}$ in H_2O.*

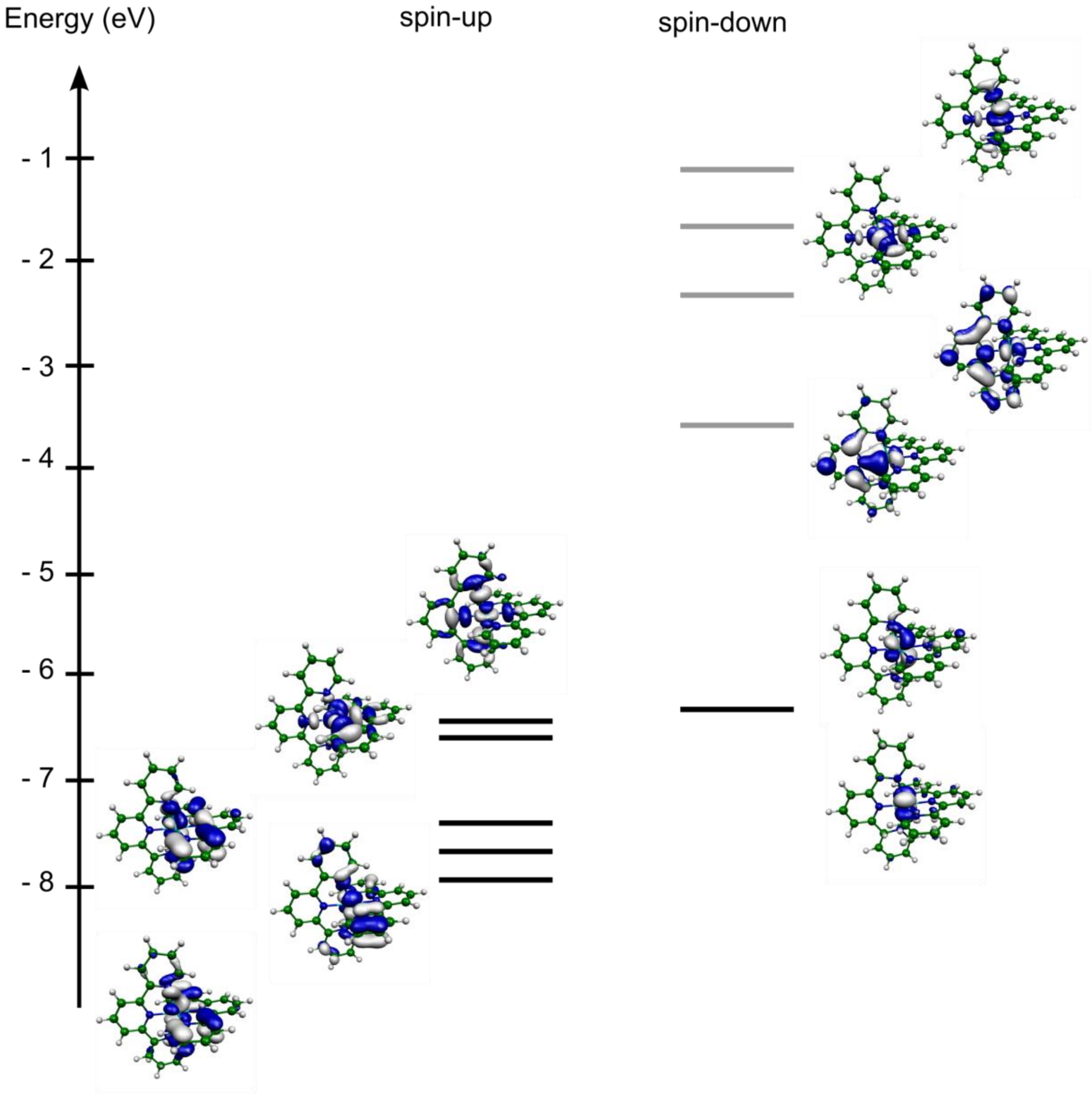

***Figure S1b**. Unrestricted spin-up(alpha)/spin-down(beta) orbitals for the quartet HS state of $[Co(terpy)_2]^{2+}$ in H_2O.*

The observation that more than 5 orbitals have a dominant Co-3d character highlights the importance of covalency in these systems.

References:

[reiher2001] DOI 10.1007/s00214-001-0300-3

[reiher2002] DOI 10.1021/ic025891l

[neese2012] DOI 10.1002/wcms.81

[papai2013] DOI 10.1039/c7sc02815f

[leshchev2018] DOI 10.1039/C7SC02815F

[szemes2020] DOI 10.1039/d0cc04467a

[klamt1993] DOI 10.1039/P29930000799

[neese2003] DOI 10.1002/jcc.10318

[neese2009] DOI 10.1016/j.chemphys.2008.10.036

[hirata1999] DOI 10.1016/S0009-2614(99)01149-5

S.I.3. Transient optical absorption spectroscopy experiments

Data acquisition

Ultrafast transient absorption (TA) measurements were carried out at the Center for Nanoscale Materials (CNM) at Argonne National Laboratory. An amplified Ti:sapphire laser system (Spectra Physics, Solstice Ace) was used to produce a 5kHz train of 1.6 mJ, 100 fs pulses at 800 nm. The 800 nm output was split 90/10 with the 90% portion used to generate the excitation pulses by pumping an optical parametric amplifier (Light Conversion, TOPAS). The 10% portion was used to generate the continuum probe after double passing a computer controlled optical delay line then focusing into a 3 mm thick sapphire crystal. An interference notch filter was used to remove the residual high-intensity 800 nm light from the continuum which left the probe beam covering 440-760 nm. The excitation beam, 0.16−0.32 μJ/pulse, was depolarized and synchronously chopped at 2.5 kHz. The excitation and probe beams were individually focused to ca. 200 μm diameter and spatially overlapped on the sample solution contained in a stirred 2 mm path length cuvette. The transmitted probe beam was recollimated then focused into a fiber-coupled spectrometer and detected with a 1D, 2048-pixel CCD array detector. The spectrometer was read out at 5 kHz producing an alternating series of T_{ON} and T_{OFF} values. The differential extinction (ΔA) was calculated as $\Delta A = -\log10(T_{ON}/T_{OFF})$ for each pair of pulses typically averaging for 2 s for each time point. The resulting data set is a three-dimensional map of spectra/time/ΔA. The stimulated Raman signal from the solvent was used to estimate the excitation and probe pulse widths which were 107 fs assuming a sech^2 pulse shape. The temporal chirp in the probe pulse was measured and corrected for by making a measurement on the neat solvent; the resulting signal was then fitted for each probe wavelength to determine the zero-delay position between pump and probe.

Analysis

The single wavelength kinetics at the maximum of the bleach (510 nm) was fitted with a single exponential decay function convoluted with the instrument response function (IRF) set as a standard Gaussian function with fwhm of 165 fs.

S.I.4 Time-resolved X-ray emission spectroscopy experiments

Time-resolved X-ray emission spectroscopy (TR-XES) measurements were performed at the Femtosecond X-ray Experiments instrument (FXE) [Galler] of the European XFEL in Schenefeld, Germany. The standard setup for femtosecond pump-probe liquid chemistry experiments was employed [Khakhulin] and the emission spectra were collected with the 16-crystal wavelength-dispersive von Hamos spectrometer (see Figure in the main text). The beamtime proposal details are available under DOI: 10.22003/XFEL.EU-DATA-002708-00.

Von Hamos spectrometer

The von Hamos spectrometer was equipped with two sets of cylindrically bent crystal analyzers of 0.5 m radius of curvature: seven Si(531) and seven Ge(111) (4-th order) for simultaneous collection of the Co Kα and Kβ emission spectra respectively. Due to large difference in the analyzer Bragg angles for the two emission lines, the spectra were focused and recorded on two different Jungfrau detectors, the Jungfrau-1M and Jungfrau-500k for the α and β lines correspondingly. The spectra from 7 crystal analyzers of each set were overlapped in the focusing and energy-dispersive directions on the respective detector to enhance signal-to-background ratio, since the background mostly originated from isotropic elastic scattering of the incoming photons. To further reduce the background and absorption of radiation in air, the path from sample environment chamber to analyzers and from analyzers to detectors was kept in Helium gas by placing a special He-filled shell with large Kapton windows. In order to convert the spectrometer dispersive axis from detector pixels to absolute energy units, the energy scale was calibrated using a scattering target at the sample position and a monochromatic X-ray beam produced by the 4-bounce Si(111) crystal monochromator ($\Delta E/E \sim 10^{-4}$) tuned to the corresponding photon energy range around Co Kα or Kβ lines.

Sample delivery

An aqueous solution of $[Co(terpy)_2]^{2+}$ with concentration of 10 mM was prepared and recirculated in closed-loop from a 40 mL sample reservoir using an HPLC pump-based system (Shimadzu LC-20AP) through a glass capillary nozzle thus forming a cylindrical jet with 100 um diameter in the pump-probe interaction region. Linear speed of the sample jet was estimated to be about 60 m/s, which ensured replacement of exposed sample volume for each pump-probe event given the size of the interaction volume and the repetition rate of the experiment (see next section). The sample jet was placed in a Helium environment chamber with Kapton window (25 um thickness) towards the emission spectrometer. No sample

degradation was observed during the measurements as monitored by reference emission spectra and by measuring UV-VIS spectra of solution. The in situ optical spectra measurements were used to keep the sample concentration constant by refilling the sample reservoir with pure water in case of solvent evaporation.

X-ray and optical laser beam conditions

The pump-probe XES experiments were conducted using 9.3 keV incoming X-ray photons and optical laser excitation with 400 nm wavelength. Femtosecond X-ray pulses from the European XFEL machine were bunched in trains of 150 pulses each with an intra-train repetition rate of 564 kHz, while the train rate was 10 Hz. Each X-ray pulse had energy of approx. 350 uJ at the sample position and the beam was focused to a round spot with 7 um diameter (fwhm) by means of Beryllium compound refractive lenses. The optical laser beam was overlapped with the X-ray beam on the jet at a crossing angle of approximately 15° in the horizontal plane. The laser beam spot size on the jet was 73 x 62 um^2 fwhm, pulse energy was set to 34 uJ (or 16 uJ for low power measurements) while pulse duration was 70 fs fwhm. This corresponds to incident laser fluence of approximately 590 mJ/cm^2 and 300 mJ/cm^2 for the high and low power measurements or, considering the molar absorptivity of solution ε=2000 1/M/cm, to 9 or 4.6 photons per absorption cross-section on the jet surface.

Despite such intense excitation, the difference XES signals measured at 500 fs delay demonstrate linear behavior. It should be noted that neither the MLCT nor the metastable quartet state absorb at 400 nm. Figure **S2** shows both Co Kα and Kβ difference signals measured with high and low excitation pulse energy of 34 uJ and 16 uJ. The high pulse energy signals were scaled by a factor of 34/16=2.12 and match well the low power measurement to within the statistical uncertainties. The signals were reduced according to the procedure described in the next section. Additionally, Figure **S3** presents a full scan pump pulse energy and the difference Kα XES signal strengths was defined as an integral of absolute difference in the emission energy range of Figure **S2-A.**

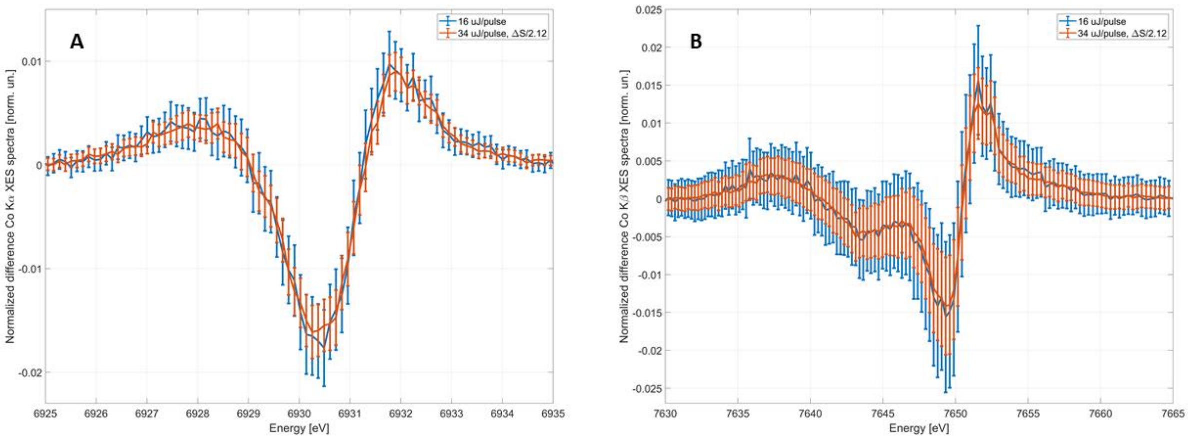

***Figure S2**. Comparison of the difference XES signals for high (red) and low (blue) excitation power conditions, A - Co Kα and B - Co Kβ spectra. The high-power data points are scaled according to the pulse energy ratio. Y-axis is in the units of relative change of the respective emission line intensity, i.e. typical maximum difference is on the order of 1-4 %.*

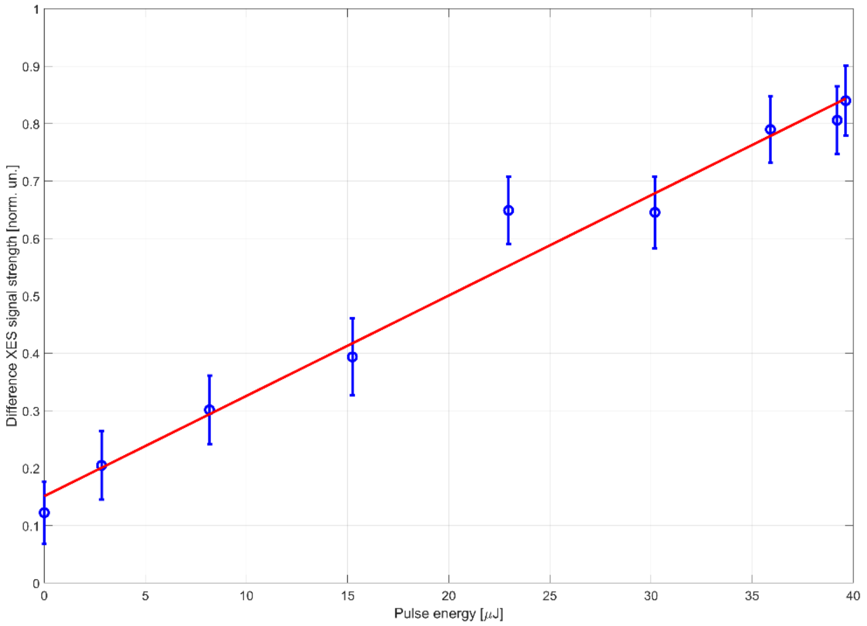

***Figure S3**. Pulse energy scan of the difference Co Kα spectra. The signal strength is determined upon integration of absolute difference signal in Figure **S2-A**.*

Like X-ray pulses, the optical laser pulses were grouped in trains of 150 pulses at 564 kHz, however the train repetition rate was kept at 5 Hz in order to realize the alternating laser-on

and laser-off conditions for every other X-ray train of pulses. Estimated temporal width of the instrument response function (IRF) was about 115 fs (fwhm) in agreement with the earlier results reported elsewhere [Khakhulin]**.**

Data acquisition, reduction and analysis

X-ray emission spectra were recorded by two Jungfrau detectors synchronized to the 10 Hz train rate, implying that spectra from every X-ray pulse train of 150 pulses were accumulated in single detector images collected in 350 us-long exposures. The Jungfrau images were corrected for the leakage current offset and gain factors per pixel considering the activated gain stage and according to the standard image correction procedure of European XFEL. Since only for every other X-ray train the sample jet was exposed to the optical laser train, sets of alternating laser-on and laser-off detector images were acquired for every setting of an experimental parameter (here pump-probe delay or optical excitation power). Experimental data were collected either in a step-wise scanning way with typical acquisition time of 1 minute per data point or in a long measurement of 10-15 min at a fixed parameter (delay) setting. The recorded detector data were sorted in laser-on and laser-off images and according to delay using the attributed unique train identifier for each image and motor position. The laser-on and laser-off images were then processed in the same way by selecting a 8-pixel wide main region of interest (pixel size is 75x75 um^2) around the emission line (Co Kα or Kβ) in the spectrometer focusing direction and summing the signal along this direction to extract 1D spectral curves. Two additional regions of interest were selected symmetrically in the images on both sides of the main region and averaged to approximate elastic scattering background in the main region, presumably linear in the focusing direction. The resulting background was summed along the focusing direction and subtracted from the main emission spectra, then the spectra were self-normalized to their total area. Based on the normalization factor the weakest spectra no percentile per px was applied to enhance S/N ratio in the normalized average. After averaging within a given parameter setting, the normalized Laser-on spectra were subtracted from the laser-off spectra to result in transient line-shapes with respective standard error of the mean. Since the characteristic features of the difference Kβ spectra are not sharp, the spectra are much oversampled by the small pixel size of the Jungfrau detector. To enhance S/N ratio the difference Kβ spectra were binned by 4 energy points essentially without any loss of energy resolution for spectral features. To produce kinetic plots from delay scans, several regions of interest were defined on the energy scale and corresponding absolute values of the transient line-shapes were integrated in these ranges indicating varying spectral sensitivity to different excited states.

Linearization of the Jungfrau detector response in the high-to-medium gain switching region

As a large dynamic range and adaptive gain detector, Jungfrau may exhibit response artifacts especially in the gain switching region. Depending on the operating conditions and detected signal levels, the gain switching region can demonstrate discontinuity as either imperfect offset correction or deviating gain constant in the medium gain stage. In order to eliminate potential influence of the gain switching artifacts on the emission spectra we performed *in-situ* characterization and correction of the response. It is important to note that in the present case such correction is only required for Kα spectra, since Kβ signals are substantially lower than the gain transition. Region on the detector where $K\alpha_{1,2}$ signals didn't exceed gain switching threshold for all recorded images, was taken as a reference (I_0). Plotting Ka_1 signal versus reference (I_0) highlights the non-linearity and required corrections. Based on the linear fitting of signals in the high and medium gain stage using large sets of data with varying signal levels, we correct response in the medium gain stage per-pixel, thus compensating detection artifacts. Further details on self-referenced pixel-based approaches for correcting gain switching artifacts are reported elsewhere [Biednov].

References

[Galler] A. Galler et al., DOI: 10.1107/S1600577519006647

[Khakhulin] D. Khakhulin et al., DOI: 10.3390/app10030995

[Biednov] M. Biednov et al., in preparation.

Transient versus static reference spectrum

Figure **S4** displays the comparison between the normalized transient signal observed for a pump-probe delay of 500 fs and the static reference trace after scaling. The scaling factor delivers the excited state fraction for this particular pump-probe delay.

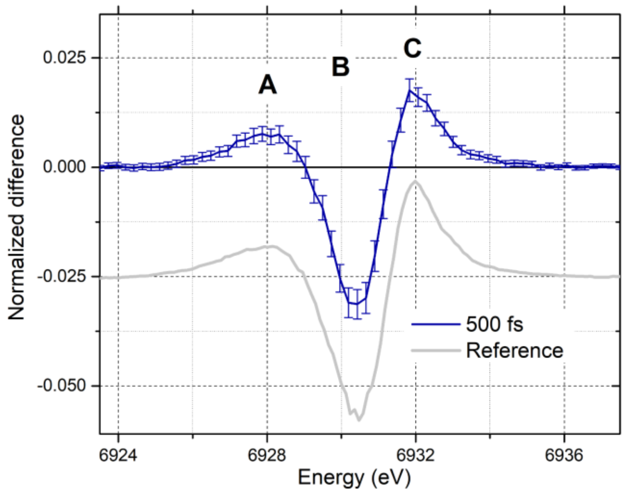

***Figure S4**. Normalized transient at 500 fs and static reference.*

S.I.5 *Ab initio* X-ray emission calculations

Ligand field splittings and magnetic parameters

Orca 5.0.1 [Neese] was used to calculate the ligand field parameters, g-matrix, and D-tensor for the DFT-optimized doublet and quartet structures. The active space comprised the seven electrons distributed in the five metal d-orbitals denoted CASSCF(7,5). To recover part of the dynamic electron correlation effects on the energies of the states, we run N-electron valence perturbation theory (NEVPT2) [Angeli] calculations on top of the CASSCF wavefunctions. The DKH2 [Hess] scalar relativistic Hamiltonian was used in all calculations, together with the DKH2 recontracted def2-TZVP basis for all elements [Pantazis]. We included all possible doublet (10) and quartet (40) states within the selected active space. The orbitals were averaged over all calculated states. The ligand field parameters and magnetic properties are listed in Table **S5**. All reported values include the NEVPT2 correction.

Table S5. *Orbital energies and g-tensor*

	Quartet	Doublet
d-orbital energies (eV)	0.0	0.0
	0.016	0.009
	0.050	0.049
	0.934	1.195
	1.280	2.116
g_x	1.766	1.994
g_y	2.423	2.057
g_z	2.882	4.106
D (cm^{-1})	119.75	
E/D	0.14	

Multiplet calculations of X-ray emission spectra

The Kα and Kβ X-ray emission spectra (XES) were calculated using the Quanty [Haverkort] library, which allows for spectroscopy calculations with user-defined semi-empirical Hamiltonians. The starting input file was generated with the Crispy graphical user interface [Crispy]. Only atomic and crystal field interactions have been considered. The atomic interactions of the isolated Co(II) ion were parameterized using the Slater integrals, Coulomb (Fk) and exchange (Gk), and the spin-orbit coupling parameters (ζ), and were calculated using the Hartree-Fock theory. To partially account for covalency effects and intra-atomic relaxation processes the values of the Slater integrals are reduced using scale factors (κ).

	$3d^7$		$1s^1 3d^8$		$2p^5 3d^8$		$3p^5 3d^8$	
	value	scale factor (κ)	value	scale factor (κ)	value	scale factor (κ)	value	scale factor (κ)
F^2_{dd}	11.605	0.6	13.347	0.6	13.422	0.6	12.706	0.6
F^4_{dd}	7.209	0.6	8.339	0.6	8.395	0.6	7.944	0.6
ζ_d	0.066	1.0	0.093	1.0	0.092	1.0	0.075	1.0
G^2_{sd}			0.072	0.6				
F^2_{pd}					7.900	0.6	13.694	0.6
G^1_{pd}					5.951	0.6	16.949	0.6
G^3_{pd}					3.386	0.6	10.328	0.6
ζ_p					9.746	1.0	1.168	1.0

Crystal field parameters were taken from the Orca calculations presented above. All parameters were varied to improve the agreement with the experimental measurements. Limiting the number of parameters that enter the XES calculation is essential as emission lines have a limited number of features. Therefore, we used the same scale factors for the atomic

parameters and the same crystal field values in the Kα and Kβ calculations for a given ground state (doublet or quartet). In a final step, theoretical spectra must be convoluted to be comparable with experimental data. Two function types are commonly used: a Gaussian to account for the experimental broadening and a Lorentzian to account for the lifetime broadening of the excited states. For the latter, we used an energy-depended full-width half-maximum (fwhm) as suggested previously [Glatzel]. The variation of the Lorentzian fwhm with energy is plotted below (Figure **S6**). As before, the broadening values were kept constant in all calculations for a given emission line to limit the number of parameters.

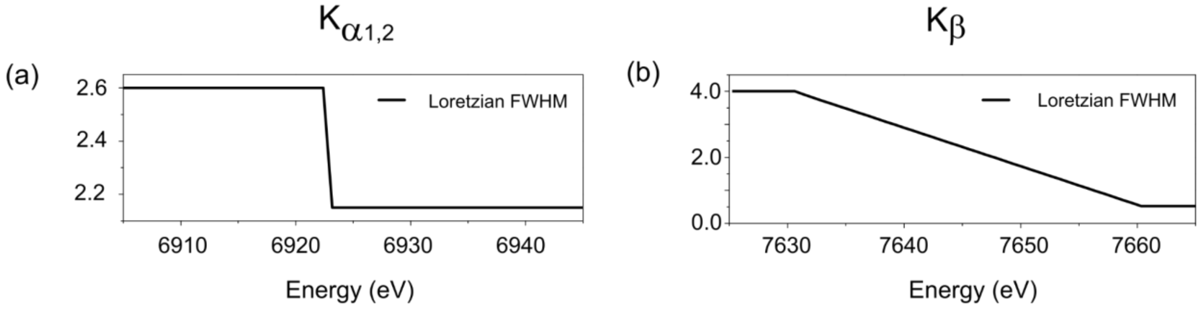

Figure S6*. Energy-dependent Lorentzian fwhm of the Lorentzian for (a) Kα1,2 and (b) Kβ.*

References

[Neese] Neese, DOI: 10.1002/wcms.1606

[Angeli] Angeli, DOI: 10.1063/1.1361246

[Hess] Hess, DOI: 10.1103/PhysRevA.33.3742

[Pantazis] Pantazis *et al.*, DOI: 10.1021/ct800047t

[Haverkort] Haverkort, DOI:10.1088/1742-6596/712/1/012001

[Crispy] Retegan, M. Crispy: v0.7.3 DOI: 10.5281/zenodo.1008184.

[Glatzel] Glatzel *et al.*, DOI:10.1103/PhysRevB.64.045109

S.I.6 Assessing the possible observation of the MLCT in the transient XES measurements

Model difference spectra

The transition (MLCT-LS) is modeled using the traces from $[Co(terpy)_2]^{3+}$ and $[Co(terpy)_2]^{2+}$

The transition (HS-LS is modeled using the traces from $[Co(bpy)_2]^{2+}$ ando $[Co(terpy)_2]^{2+}$

The intermediate traces are modeled by (x) $[Co(terpy)_2]^{3+}$ + (1- x) $[Co(bpy)_2]^{2+}$ - $[Co(terpy)_2]^{2+}$ for x ranging from 0.2 (orange trace) to 0.8 (blue trace), as shown in Figure **S7**.

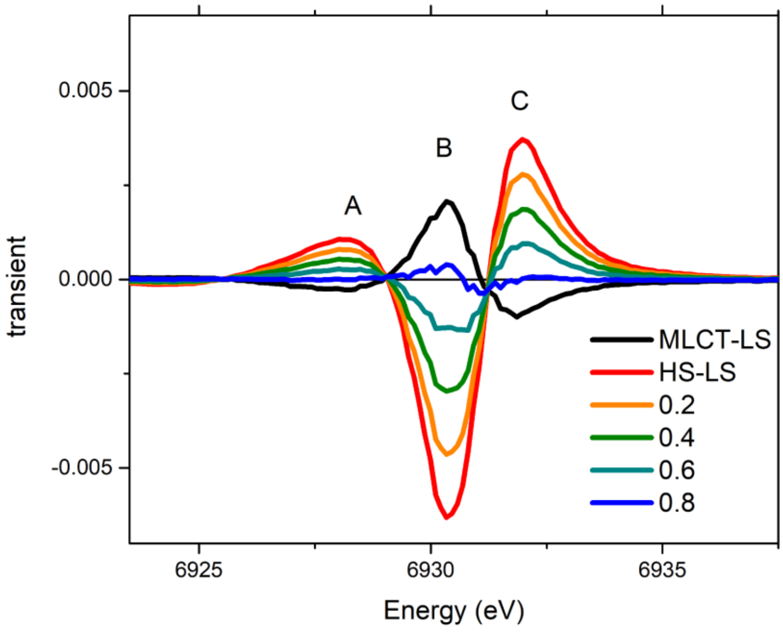

Figure S7. *Model difference spectra for variable fraction of MLCT population.*

S.I.7 Fitting of the energy-resolved Kα_1 X-ray kinetics

All energy-resolved kinetics were fitted with the equation of type:

$$S_{xes}(t) = A \cdot \frac{1}{2}\left(erf\left(\frac{t-t0}{\sqrt{2}\,\sigma}\right)+1\right)\cdot\left(2-e^{(t-t0)/\tau}\right)$$

where *erf* is the Gaussian error function with width of σ and the argument (t) offset of t_0.

The kinetics data with experimental errors were fitted using the sum of least-squares method in the Origin software. The width of the signal step function was fixed constant for all fits to the best fit for spectral area B as it provides the smallest uncertainty of this parameter, σ = 71 fs (rms) or 167 fs fwhm. Three models are considered: Model I with fixed exponential risetime and varied t_0, Model II with fixed t_0 and varied risetime and Model III with varied t_0 and the risetime. The results are summarized in Figures **S8-S10** and Table **S11**, **S12** below.

Model I

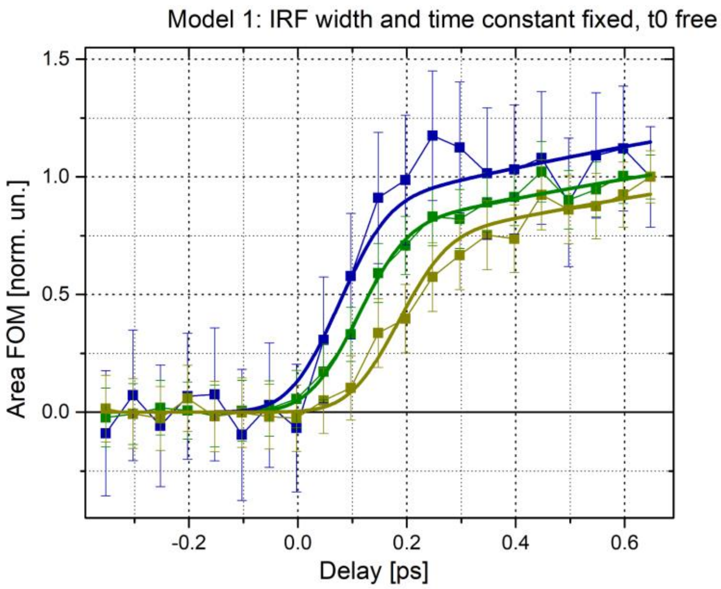

***Figure S8**. Energy-resolved kinetics and best-fit curve using Model I.*

Model II

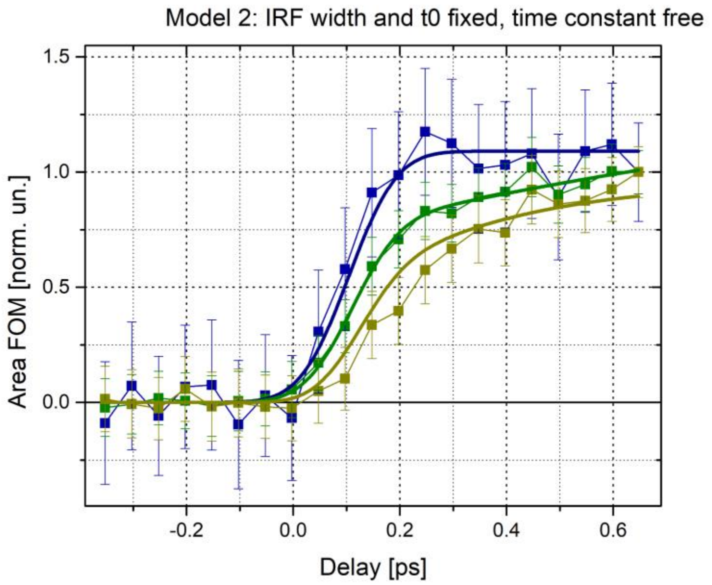

Figure S9. Energy-resolved kinetics and best-fit curve using Model II.

Model III

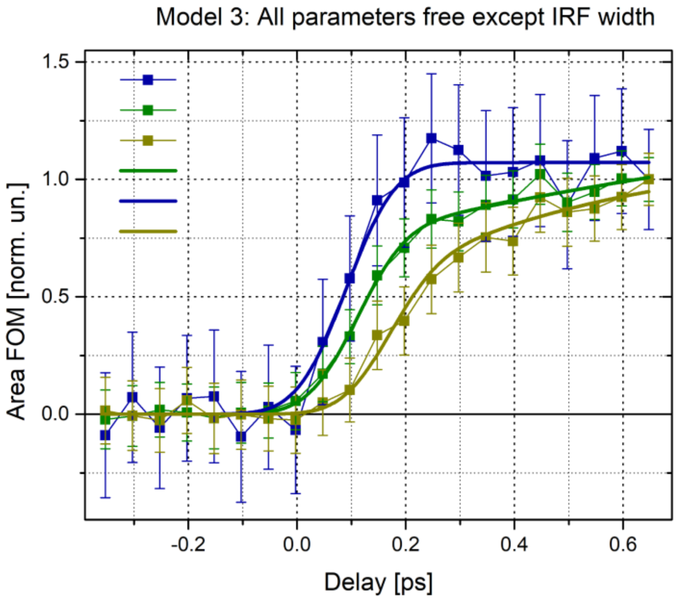

Figure S10. *Energy-resolved kinetics and best-fit curve using Model III.*

Table S11. *Summary of the best-fit parameters for Kα and Kβ kinetics acquired simultaneously (fixed 115 fs IRF for Kα and Kβ).*

	t_0 [ps]	τ [ps]	**R^2; Red.X^2**
**Kα**	0.0870 +/- 0.005	0.270 +/- 0.08	0.995; 0.136
**Kβ**	0.130 +/- 0.02	0.320 +/- 0.40	0.988; 0.671

Table S12*. Summary of the best-fit parameters for Model I, Model II and Model III (fixed 115 fs IRF for Kα and Kβ).*

	$t_{0,1}$, $t_{0,2}$, $t_{0,3}$ [ps]	τ_1, τ_2, τ_3 [ps]	**R^2 Red.X^2**
**Model I (*τ fixed*)**	$t_{0,1}$ = 0.471 +/- 0.002<br>$t_{0,2}$ = 0.079 +/- 0.004<br>$t_{0,3}$ = 0.15 +/- 0.01	τ = 0.23	R^2 = 0.957<br>Red. X^2=0.165<br>R^2 = 0.995<br>Red. X^2= 0.074<br>R^2 = 0.983<br>Red. X^2= 0.150
**Model II (*t0 fixed*)**	t_0 = 0.079	τ_1 = 60 +/- inf<br>τ_2 = 0.23 +/- 0.05<br>τ_3 = 0.3 +/- 0.3	R^2 = 0.977;<br>Red. X^2= 0.089<br>R^2 = 0.995<br>Red. X^2= 0.074<br>R^2 = 0.941<br>Red. X^2 = 0.529
**Model III**	$t_{0,1}$ = 0.09 +/- 0.01<br>$t_{0,2}$ = 0.079 +/- 0.005<br>$t_{0,3}$ = 0.15 +/- 0.02	τ_1 = 1000 +/- inf<br>τ_2 = 0.23 +/- 0.05<br>τ_3 = 0.36 +/- 0.49	R^2 = 0.976;<br>Red. X^2= 0.096<br>R^2 = 0.995;<br>Red. X^2= 0.078<br>R^2 = 0.985;

			Red. X^2= 0.141

S.I.8 DFT structure at the crossing between the PECs of the lowest doublet and lowest quartet states

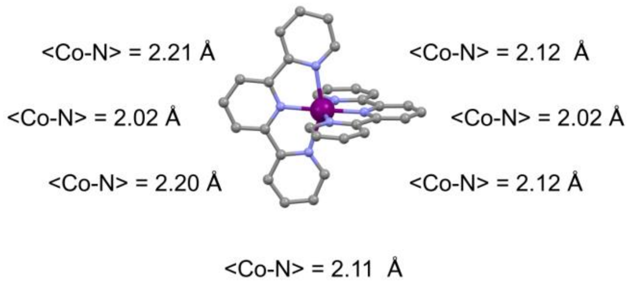

***Figure S13**. DFT structure adopted by [Co(terpy)$_2$]$^{2+}$ in H_2O at the crossing between the PECs of the lowest doublet and quartet states. The individual Co-N bonds and their average are indicated.*

S.I.9 Comparison of the ultrafast photoswitching parameters in spin-crossover molecules

Table S14 summarizes the lifetime of the metastable spin-state (MS) populated upon photoexcitation of the ground state (GS) for several homoleptic spin-crossover molecular complexes with FeN_6 first-coordination sphere. The measurements are conducted with different X-ray techniques, namely X-ray Absorption Spectroscopy (XAS), X-ray Emission Spectroscopy (XES) and X-ray Diffuse Scattering (XDS) achieving picosecond temporal resolution. It should be noted that the lifetimes are solvent-dependent (see individual references for experimental details).

***Table S14**. Lifetimes of the metastable spin-state for several homoleptic spin-crossover molecules with FeN_6 first-coordination sphere.*

**Complex**	**Transition GS/MS**	**Lifetime (ps)**	**Technique**	**Reference**
$[Fe(bpy)_3]^{2+}$	HS/LS	650	XAS	(Gawelda)
$[Fe(bpy)_3]^{2+}$	HS/LS	594	XAS	(Haldrup1)
$[Fe(bpy)_3]^{2+}$	HS/LS	503	XES	(Haldrup1)
$[Fe(bpy)_3]^{2+}$	HS/LS	657	XDS	(Haldrup1)
$[Fe(bpy)_3]^{2+}$	HS/LS	1370	XAS	(Canton)
$[Fe(phen)_3]^{2+}$	HS/LS	690	XAS	(Nozawa)
$[Fe(tren(py)_3)]^{2+}$	HS/LS	60000	XAS	(Huse)
$[Fe(terpy)_2]^{2+}$	HS/LS	4215	XAS	(Canton)
$[Fe(terpy)_2]^{2+}$	HS/LS	2610	XAS	(Vanko)
$[Fe(dcpp)_2]^{2+}$	HS/LS	270	XAS	(Britz1)

Table S15 summarizes the switching time from the ground state (GS) to the metastable spin-state (MS) populated upon photoexcitation for several homoleptic spin-crossover molecular

complexes with FeN_6 first-coordination sphere. The measurements are conducted with different X-ray techniques achieving femtosecond resolution. The timescales associated to the sequential steps are also given, when delivered by the experiment.

***Table S15**. Lifetimes of the metastable spin-state for several homoleptic spin-crossover molecules with FeN_6 first-coordination sphere.*

Complex	Transition GS/MS	Timescale (fs)	Technique	Reference
$[Fe(bpy)_3]^{2+}$	LS/HS	150	XAS	(Bressler)
$[Fe(bpy)_3]^{2+}$	LS/HS	160	XAS	(Lemke1)
$[Fe(bpy)_3]^{2+}$	LS/HS	120<br>1600	XAS	(Lemke2)
$[Fe(bpy)_3]^{2+}$	LS/HS	150<br>70	XES	(Zhang1)
$[Fe(bpy)_3]^{2+}$	LS/HS	150	XES	(Haldrup2)
$[Fe(bpy)_3]^{2+}$	LS/HS	600	XDS	(Haldrup2)
$[Fe(bpy)_3]^{2+}$	LS/HS	110<br>72	XES/XDS	(Kjaer)
$[Fe(terpy)_2]^{2+}$	LS/HS	100	XAS	(Britz2)
$[Fe(phen)_3]^{2+}$	LS/HS	170<br>39	XAS	(Zhang2)

References

(Bressler) Femtosecond XANES Study of the Light-Induced Spin crossover Dynamics in an Iron (II) Complex, DOI: 10.1126/science.1165733.

(Britz1) Using Ultrafast X-ray Spectroscopy To Address Questions in Ligand-Field Theory: The Excited State Spin and Structure of $[Fe(dcpp)_2]^{2+}$, DOI: 10.1021/acs.inorgchem.9b01063.

(Britz2) Resolving Structures of Transition Metal Complex Reaction Intermediates with Femtosecond EXAFS, DOI: 10.1039/C9CP03483H

(Canton) Probing the Anisotropic Distortion of Photoexcited Spin Crossover Complexes with Picosecond X-ray Absorption Spectroscopy, DOI: 10.1021/jp5003963.
(Gawelda) Structural Determination of a Short-Lived Excited Iron(II) Complex by Picosecond X-Ray Absorption Spectroscopy, DOI: 10.1103/PhysRevLett.98.057.
(Haldrup1) Guest–Host Interactions Investigated by Time-Resolved X-ray Spectroscopies and Scattering at MHz Rates: Solvation Dynamics and Photoinduced Spin Transition in Aqueous $Fe(bipy)_3^{2+}$, DOI: 10.1021/jp306917x.
(Haldrup2) Observing Solvation Dynamics with Simultaneous Femtosecond X-ray Emission Spectroscopy and X-ray Scattering, DOI: 10.1021/acs.jpcb.5b12471.
(Huse) Femtosecond Soft X-ray Spectroscopy of Solvated Transition-Metal Complexes: Deciphering the Interplay of Electronic and Structural Dynamics, DOI: 10.1021/jz200168m.
(Kjaer) Finding Intersections between Electronic Excited State Potential Energy Surfaces with Simultaneous Ultrafast X-ray Scattering and Spectroscopy, DOI: 10.1039/c8sc04023k.
(Lemke1) Femtosecond X-ray Absorption Spectroscopy at a Hard X-ray Free Electron Laser: Application to Spin Crossover Dynamics, DOI: 10.1021/jp312559h.
(Lemke2) Coherent Structural Trapping through Wave Packet Dispersion during Photoinduced Spin State Switching; DOI: 10.1038/ncomms15342.
(Nozawa) Direct Probing of Spin State Dynamics Coupled with Electronic and Structural Modifications by Picosecond Time-Resolved XAFS, DOI: 10.1021/ja907460b.
(Vanko) Detailed Characterization of a Nanosecond-Lived Excited State: X-ray and Theoretical Investigation of the Quintet State in Photoexcited $[Fe(terpy)_2]^{2+}$, DOI: 10.1021/acs.jpcc.5b00557.
(Zhang1) Tracking Excited-State Charge and Spin Dynamics in Iron Coordination Complexes, DOI: 10.1038/nature13252.
(Zhang2) Tracking the Metal-Centered Triplet in Photoinduced Spin Crossover of $Fe(phen)_3^{2+}$ with Tabletop Femtosecond M-Edge X-ray Absorption Near-Edge Structure Spectroscopy, DOI: 10.1021/jacs.9b07332.
